# A single nucleotide polymorphism in primary-microRNA-146a reduces the expression of mature microRNA-146a in patients with Alzheimer's disease and is associated with the pathogenesis of Alzheimer's disease

**DOI:** 10.3892/mmr.2015.3968

**Published:** 2015-06-22

**Authors:** BIN ZHANG, AIHONG WANG, CUIPING XIA, QUNFENG LIN, CHUNFU CHEN

**Affiliations:** 1 Department of Neurology, Shandong Provincial Hospital, Shandong University, Jinan, Shandong 250012, P.R. China; 2 Department of Neurology, Shanghai Fengxian Central Hospital, Shanghai 201499, P.R. China

**Keywords:** polymorphism, Alzheimer's disease, microRNA, microRNA-146a, Toll-like receptor 2, innate immunity

## Abstract

Alzheimer's disease (AD) is a progressive neurodegenerative disorder and is the most common form of dementia among the aging population. Although the incidence of the disease continues to increase, no cure has been developed. Effective treatment is restricted not only due to the lack of curative medicine, but also due to limited understanding of the underlying mechanisms and the diffculties in accurately diagnosing AD in its earliest stages prior to clinical symptoms. Micro (mi) RNAs (miR) have gained increasing attention in the investigation of neurodegenerative diseases. Previous reports have demonstrated that deregulation of miR-146a-5p is associated with the pathogenesis of human AD. In the present study, the coding region of primary (pri)-miR-146a in patients with AD was scanned and the rare C allele of rs2910164 was found to be associated with AD. Using reverse transcription quantitative polymerase chain reaction, it was demonstrated that site variation reduced the expression of mature miR-146a-5p. Notably, a reduction in the expression of miR-146a-5p led to less effcient inhibition of target genes, including Toll-like receptor (TLR)2, which is important in the pathogenesis of AD. Biological function investigations in RAW264.7 cells indicated that, compared with the G allele, the rare C allele upregulated the expression of tumor necrosis factor-α following stimulation with β-amyloid. These findings suggested that one common polymorphism in pri-miR-146a may contribute to the genetic predisposition to AD by disrupting the production of miR-146a-5p and affecting the expression and function of TLR2.

## Introduction

Alzheimer's disease (AD) is a common, age-associated neurodegenerative disorder, which is characterized by a loss of synapses and neurons, intracellular neurofibrillary tangles and the formation of extracellular amyloid plaques (1). The classical hypothesis for the cause of AD is the aberrant amyloid protein deposition of amyloid β (Aβ)42 (2). The etiology of AD is complex and is composed of genetic and environmental factors (3,4). There is evidence indicating that dysfunctions of neuro-immune networks also contribute to the pathogenesis of AD (5,6).

Increasing evidence suggests that microglia are important in the pathophysiology of AD, and Toll-like receptor (TLR)2 is one of the pattern recognition receptors expressed in microglia, which was originally identified based on their response to invading microorganisms (7). In mouse models of AD, microglia are activated and recruited to the deposits of pathogenic Aβ, where they subsequently damage neurons (8–10). Deficiencies in TLR2 and TLR4 in cultured microglia are significantly reduced following Aβ-triggered inflammatory activation (11,12). TLR2 knockout in a mouse model of AD decreased the deposition of cerebral Aβ (13). Additionally, overexpression of TLR2 has been observed in patients with AD and demonstrates that TLR2 is important in Aβ-triggered inflammatory activation and Aβ phagocytosis (14).

MicroRNAs (miRs) are a group of negative regulators of gene expression, which repress gene expression by directly binding the 3′-untranslated region (UTR) of mRNA. miRs are important in maintaining normal physiological conditions in the human body, and abnormal expression of miR has been associated with several human diseases, including psychiatric disorders and certain types of malignant cancer (15–17). The dysfunction of miR in neurodegenerative disorders is becoming increasingly recognized. miR-146a has been demonstrated to be important in AD, with aberrant expression of miR-146a having been identified in transgenic mouse models of AD and in human AD brain cells (18,19). Therefore, the present study aimed to investigate whether single nucleotide polymorphisms (SNPs) or mutations in the miR-146a coding region were associated with the pathogenesis of AD.

Primary (pri)-miR-146a was sequenced in the genomic DNA of 103 patients with AD. The effect of a polymorphism site on the expression of mature miR-146a was examined and a novel miR-146a target gene was confirmed. The biological function of this site on miR-146a target genes expression and the immune response to Aβ42 was also investigated.

## Materials and methods

### DNA collection and genotyping

Venous blood samples from 103 patients with AD (age range, 56–85 years; mean ± standard deviation, 68.32±8.12) and 206 healthy individuals (54–88 years; 69. 03 ±7.43) were obtained from Shanghai Fengxian Central Hospital (Shanghai, China). All the patients were from the Han population and their ancestries were from the North China plain area (Beijing, Shandong and Hebei province). The study was approved by the ethics committee of the Department of Neurology, Shanghai Fengxian Central Hospital. Written informed consent was obtained from the patient's family.

Venous blood (5 ml) was collected from patient and control individuals upon their first admission into the hospital. To harvest cell-free serum, the blood was drawn into sterile tubes (BD Biosciences, Franklin Lakes, NJ, USA) without anticoagulant and left in a standing position for 20 min. The samples were centrifuged at 1,500 x g for 10 min at 20°C and the supernatant serum was removed and stored immediately at -80°C, until further analysis.

The DNA from the blood samples was extracted using a TIANamp Blood DNA kit (Tiangen, Beijing, China). The DNA specimens were amplified by using standard PCR techniques. A total of 50 ng DNA from each sample was used for the PCR reaction. The Pfu DNA polymerase was purchased from Tiangen. The cycling conditions were as follows: 95°C for 10 min; 95°C for 30 sec followed by 60°C for 1 min for 30 cycles. The primer sequences were as follows: Forward, 5′-GGTCTCCTCCAGATGTTTATAACTC-3′ and reverse 5′-GAACCCTGCTTAGCATAGAATTC-3′. The PCR products were sequenced in forward direction with the ABI 3730xl sequencing platform (Applied Biosystems, Foster City, CA, USA) by BGI-GBI Biotech Co., Ltd. (Beijing, China). The sequencing results were analyzed using DNAMAN version 7,0,2,176 (Lynnon Corporation, San Ramon, CA, USA) and Chromas Lite version 2.22 (Technelysium Pty Ltd., South Brisbane, Queensland, Australia) software.

### Cell culture

The RAW264.7, A549 and HEK293T cells (China Infrastructure of Cell Line Resources, Beijing, China) were cultured in Dulbecco's modified Eagle's medium (Corning Incorporated, Corning, NY, USA) containing 10% fetal bovine serum (Hyclone, Logan, UT, USA), 100 U/ml penicillin and 10 mg/ml streptomycin (Hyclone). All the cells were maintained at 37°C in an atmosphere of 5% CO_2_.

### miR-146a expression vectors

To construct the miR-146a expression vectors, fragments (432 nt) corresponding to the pri-miR-146a and its flanking regions (previously determined to have the two genotypes) were amplified from the cDNA and cloned into the pcDNA3.1 vector (Invitrogen Life Technologies, Carlsbad, CA, USA). The sequences of the vectors were confirmed by direct sequencing and the only difference was in the mutation site. The miR-146a expression vectors were transfected into the cells using Lipofectamine 2000 (Invitrogen Life Technologies), according to the manufacturer's instructions. An empty pcDNA3.1 vector was used as a control.

### RT-qPCR

RT-qPCR analysis was used to determine the relative expression of miR-146a-5p. The total RNA was extracted from cells using TRIzol reagent (Invitrogen Life Technolgies) according to the manufacturer's instructions. The expression of miR-146a-5p was detected by TaqMan miRNA RT-Real Time PCR (Applied Biosystems). Single-stranded cDNA was synthesized using a TaqMan MicroRNA Reverse Transcription kit (Applied Biosystems) and then amplified using TaqMan Universal PCR Master mix (Applied Biosystems) together with miRNA-specific TaqMan MGB probes (Applied Biosystems) targeting miR-146a-5p (Applied Biosystems). The U6 small nuclear RNA (Applied Biosystems) was used for normalization. The experiments were performed using the ABI 7300 PCR thermal cycler (Applied Biosystems). The cycling conditions for the qPCR were as follows: 95°C for 10 min, 95°C for 15 sec followed by 60°C 1 min for 40 cycles. The samples in each group were measured in triplicate and the experiment was repeated at least three times for the detection of miR-146a-5p. For serum miR-146a detection, miR-16 (Applied Biosystems) was used for normalization. Total RNA was extracted from a 200 *µ*l serum sample from each participant using TRIzol LS reagent (Invitrogen Life Technologies) according to the manufacturer's instructions. The serum miR-146a and miR-16 level was detected using a specific primer and probe (Applied Biosystems) as described above.

### Dual luciferase assay

To generate a luciferase reporter vectors, full-length TLR2 3′-UTR (828 bp) was cloned downstream of the firefly luciferase coding region in the pmirGLO vector (Promega Corporation, Madison, WI, USA). The sequences of primers for TLR2 3′-UTR amplification were as follows: Forward, 5′-CTCGAGGTTCCCATATTTAAGACCAG-3′ and reverse, 5′-TCTAGATTCTCATCCTGTAAAGTTTAA TAGG-3′. The PCR cycling conditions were as follows: 95°C for 10 min, 95°C for 15 sec, 61°C for 30 sec, then 72°C for 1 min. A total of 50 ng genomic DNA was used for each PCR reaction. For the luciferase reporter assays, the HEK293T cells were seeded into 48-well plates (Corning Incorporated) at a density of 3×10^4^ cells/well. miR-146a expression vector, miR-146a mimic or miR-146a inhibitor (GenePharma Co., Ltd., Shanghai, China) were co-transfected with a luciferase reporter vector using lipofectamine 2000 (Invitrogen Life Technologies). After incubation for 4 h at 37°C, the medium was discarded and replaced with fresh DMEM medium for a further 48 h culture. The cells were harvested and assayed using a Dual-Luciferase assay (Promega Corporation). Each treatment was performed in triplicate in three independent experiments. The data are expressed as the relative luciferase activity (firefly luciferase/*Renilla* luciferase).

### Western blotting

The cells were collected by centrifugation at 1,000 x g and then resuspended in lysis buffer for 10 min to ensure complete lysis. The cells were centrifuged at 12,000 x g for 10 minutes at 4°C and the supernatant was harvested into a fresh tube. The protein quantities were detected using a bicinchoninic protein assay kit, according to the manufacturer's instructions. The protein extracts were boiled in 50 *µ*l 2X SDS/β-mercaptoethanol sample buffer (Sigma-Aldrich) and 30 *µg* samples were loaded into each lane of 8% polyacrylamide gels (Sigma-Aldrich). The proteins were separated by electrophoresis and were subsequently blotted onto polyvinylidene fluoride membranes (Amersham Pharmacia Biotech, St. Albans, UK) by electrophoretic transfer. The membrane was incubated with rabbit anti-TLR2 monoclonal antibody (Abcam, Cambridge, MA, USA; cat. no. ab108998; 1:1,000) or mouse anti-β-actin monoclonal antibody (Santa Cruz Biotechnology, Inc., Santa Cruz, CA, USA; cat. no. sc-58673; 1:1,000) for 1 h at 37°C. The specific protein-antibody complex was detected using horseradish peroxidase-conjugated goat anti-rabbit (Santa Cruz Biotechnology Inc.; cat. no. sc-2004; 1:5,000) or rabbit anti-mouse IgG (Santa Cruz Biotechnology Inc.; cat. no. sc-358920; 1:5,000). Detection of a chemiluminescence reaction was performed using an enhanced chemiluminescence kit (Pierce Biotechnology). The β-actin signal was used as a loading control.

### Aß42 challenge and ELISA detection of tumor necrosis factor (TNF)-α

The RAW264.7 cells (2×10^5^/well) were plated into 48-well plates and treated with 10 *µ*M aggregated Aβ42 (AnaSpec, Fremont, CA, USA) for 24 h. The supernatants were collected for the detection of TNF-α using a mouse TNF-α instant ELISA kit (eBioscience, San Diego, CA, USA).

### Statistical analysis

The data were analyzed using SPSS 16.0 statistical software (SPSS, Inc., Chicago, IL, USA). Statistical significance was determined using Student's t-test for two-group analyses and a Mann-Whitney U test for the tissue levels of miR-146a. The results of the genotype frequency and polymorphism distribution were analyzed using the χ^2^ test. P<0.05 was considered to indicate a statistically significant difference.

## Results

### Genotypes and risk of AD

Precursor (pre)-miR-146a is a 99 nt RNA segment and four single nucleotide polymorphisms (SNPs) exist in this region, according to the dbSNP database (build 137; http://www.ncbi.nlm.nih.gov/). To investigate whether there is an association between the nucleotide variants of pre-miR-146 and the pathogenesis of AD, the coding region of pri-miR-146a in 103 patients with AD and in 206 healthy individuals was scanned. Although no novel sequence alterations were detected, the rare C allele of rs2910164 was found to be associated with AD (OR=1.50, 95% CI=1.03–2.17; Table I).

### C allele reduces the expression of miR-146a in vitro and in vivo

**Figure 1:**
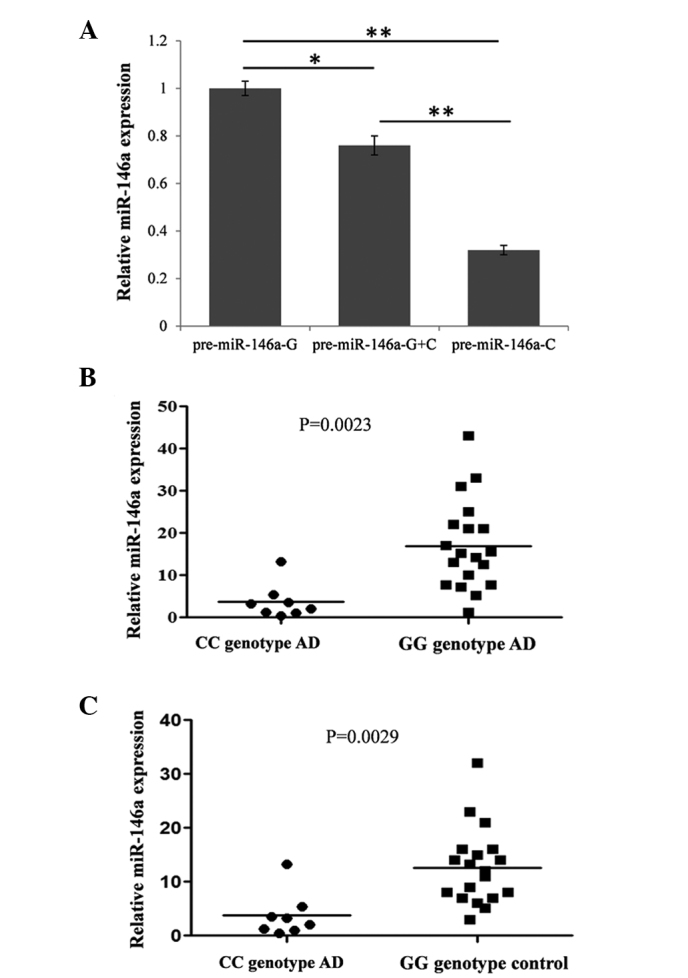
Rare C allele of rs2910164 reduces the expression of miR-146a in transiently transfected cells and patient samples. (A) Levels of mature miR-146a in cells transfected with pri-miR-146a-GG, pri-miR-146a-CC or pri-miR-146a GG and CC by reverse transcription quantitative polymerase chain reaction. The U6 small nuclear RNA was used as a normalization control. The results were analyzed using Student's t-test. Results are expressed as the mean ± standard deviation. (B) Serum miR-146a quantity compared between different genotypes of patients with AD. The serum miR-146a level between two genotypes of patients with AD were analyzed by Student's t-test. (C) Serum miR-146a quantity compared between the CC genotype of patients with AD and the GG genotype of healthy controls. The serum miR-146a level between the patients with AD of the CC genotype and GG genotype controls were analyzed by Student's t-test. (^*^P<0.05 and ^**^P<0.01). AD, Alzheimer's disease; pri-miR, primary microRNA.

Previous studies have reported that rs2910164 within the pre-miR-146a sequence reduces the levels of pri- and mature miR-146a from the C allele, compared with the allele G (1.9- and 1.8-fold, respectively) (20). The present study detected the levels of mature miR-146a in different genotypic pri-miR-146a expression vectors in transiently transfected HEK293T cells by RT-qPCR. As expected, the C allele reduced the expression of mature miR-146a to 32% compared with allele G (Fig. 1A).

To understand whether this SNP affected the expression of miR-146a *in vivo*, the expression of miR-146a was compared between the GG and CC genotypes in the serum of patients with AD and healthy controls by RT-qPCR. The expression of miR-16 was used as an internal control. The results demonstrated that the expression of miR-146a in the serum of CC genotype patients with AD was significantly downregulated compared with the GG genotype patients with AD (Fig. 1B) and the GG genotype healthy individuals (Fig. 1C).

### Expression of TLR2 is repressed by miR-146a

The function of miRs are predominantly reflected in the repression effect on their target genes. To investigate the effect of a reduced expression of miR-146a on the pathogenesis of AD, miR-146a target genes were predicted using the online bioinformatics tool, miRanda (21). This identified that TLR2, the upregulation of which is associated with triggering neuro-inflammatory activation and the pathogenesis of AD, may be a target gene of miR-146a (7).

**Figure 2:**
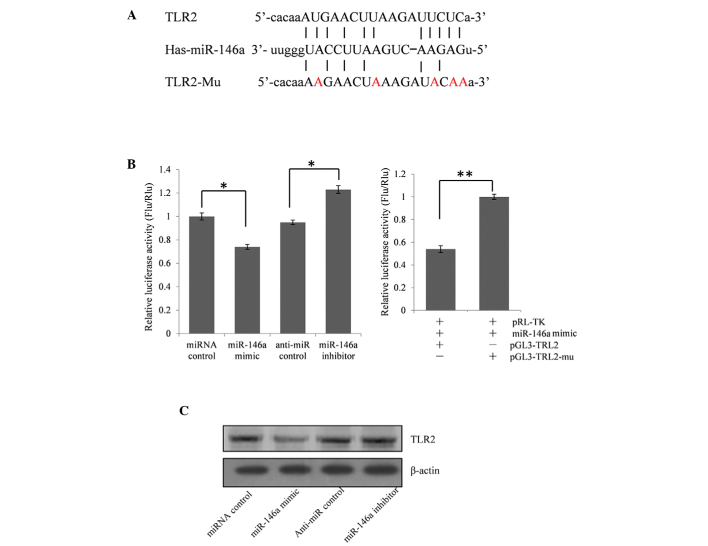
Expression of TLR2 was repressed by miR-146a. (A) Predicted miR-146a binding site in the TLR2 3′-UTR. (B) Confirmation that TLR2 is a target gene of miR-146a. HEK293T cells were co-transfected with miR control, miR-146a mimic, anti-miR control or miR-146a inhibitor and pGL3-TLR2 for the dual-luciferase assay. pRL-TK containing Rlu was co-transfected for data normalization (left). Mutation analysis of the miR-146 binding sites. Mutations in five nucleotides of the miR-146a binding site was generated (pGL3-TLR2-Mu) and the luciferase activity was significantly increased compared with wild-type TLR2 (right). The data from the two independent groups were analyzed by Student's t-test. The results are expressed as the mean ± standard deviation. (C) Protein expression of TLR2 in miR-146a mimic or inhibitor-treated A549 cells was detected by western blotting (*P<0.05 and **P<0.01). TLR, Toll-like receptor; miR, microRNA; mu, mutated; Flu, firefly luciferase; Rlu, *Renilla* luciferase.

To validate whether TLR2 is an miR-146a target gene, the full-length 828 bp segment of TLR2 3′-UTR was cloned downstream of the firefly luciferase reporter gene in the pGL3 control vector (designated as pGL3-TLR2) for the dual luciferase assay. The HEK293T cells were co-transfected with pGL3-TLR2 and a miR-146a mimic or inhibitor (Fig. 2B). The luciferase activity was reduced significantly by ~26.0% (P<0.05) in the presence of miR-146a, compared with the control. Furthermore, the luciferase activity was significantly upregulated by ~29.5% (P<0.05) following treatment with the miR-146a inhibitor, compared with the anti-miR control. These results indicated that miR-146a targeted the 3′-UTR of TLR2, leading to the change of firefly luciferase translation.

A seed sequence mutation clone was used to further confirm the binding site for miR-146a (Fig. 2A). The vector contains putative miR-146a binding regions in the 3′-UTR of TLR2, with five mutant nucleotides (designated as pGL3-TLR2-Mu). This vector was used and the wild-type TRL2 vector was used as a control. The histogram (Fig. 2B) shows that the enzyme activity was increased ~46.1% in cells co-transfected with the miR-146a mimics and pGL3-TLR2-Mu compared with pGL3-TLR2 (P<0.01). These data indicated that miR-146a suppressed the expression of TLR2 through binding to the seed sequence at the 3′-UTR of TLR2, and TLR2 may be a direct target of miR-146a.

### miR-146a regulates the endogenous expression of TLR2 in A549 cells

As TLR2 was identified as a target gene for miR-146a, whether miR-146a regulated the endogenous expression of TLR2 was examined. The A549 cells were transfected with either an miR-146a mimic or an inhibitor to determine whether the dysregulation of the expression of miR-146a affected the endogenous expression of TLR2. Compared with the corresponding control, the protein expression of TLR2 was significantly suppressed by the miR-146a mimic and was upregulated by the miR-146a inhibitor (Fig. 2C).

### Impact of rare C allele on the expression of TLR2

**Figure 3:**
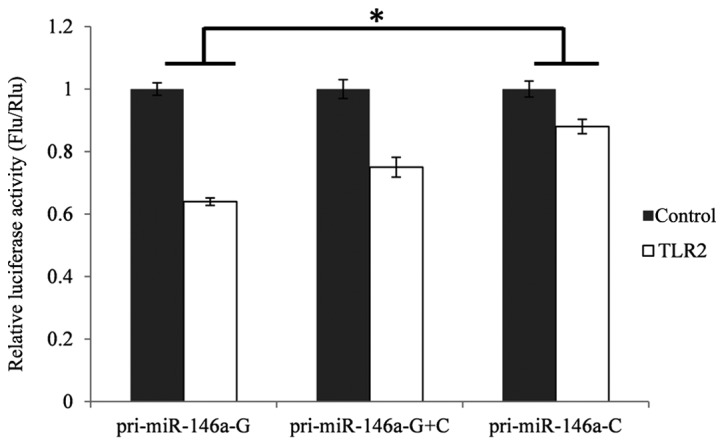
Effect of rs2910164 on the expression of TLR2. The relative luciferase activity of the reporter vector with 3′-UTR of TRL2 containing the investigated samples or deleted target sites (controls) were detected in the presence of pri-miR-146a-GG or pri-miR-146a-CC. The results from different groups were analyzed using multiple comparison/post-hoc tests of ANOVA P<0.05 was considered to indicate a statistically significant difference (^*^P<0.05). The results are expressed as the mean ± standard deviation. TLR, Toll-like receptor; pri-miR, primary microRNA; Flu, firefly luciferase; Rlu, *Renilla* luciferase.

To investigate the functional consequences of disturbed expression of miR-146a on its target genes, the TLR2 3′-UTR dual luciferase assay system was used. These reporter constructs were transiently transfected into the HEK293T cells, together with an expression plasmid containing the pri-miR-146a of either genotype. The results were analyzed using multiple comparison/post-hoc tests of analysis of variance (ANOVA) Levene's test was used to assess the variance in homogeneity, which is a pre-condition for parametric tests, including t-tests and ANOVA. The results revealed that the variances were homogeneous in TLR2 (P=0.24). As shown in Fig. 3, the activity of firefly luciferase was separately decreased by 23.9% (P<0.05) in the cells co-transfected with TLR2 and pcDNA3.1-miR146a-GG or pcDNA3.1-miR-146a-CC, compared with the control.

### Rare C allele in pri-miR-146a upregulates the production of TNF-α in RAW264.7 cells following stimulation with Aß42

**Figure 4:**
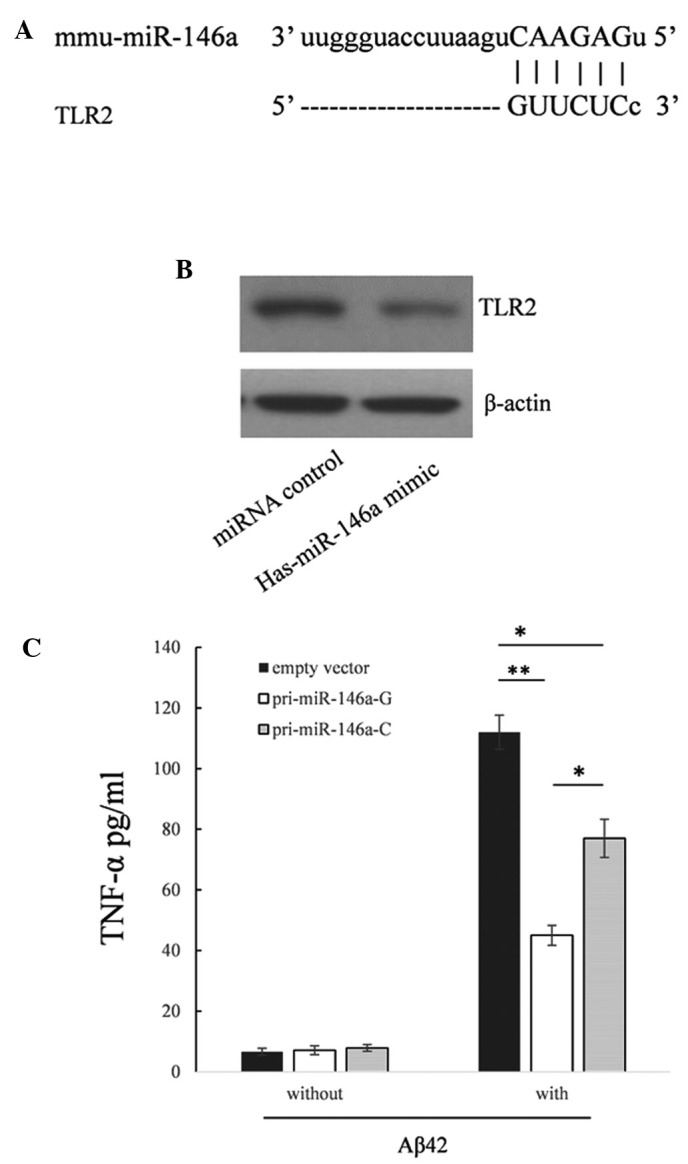
Rare C allele in pri-miR-146a upregulates the production of TNF-α in RAW264.7 cells following Aβ42 stimulation. (A) The predicted interaction between mouse miR-146a and mouse TLR2 3′-UTR. (B) Mouse expression of TLR2 can be repressed by human miR-146a. RAW264.7 cells were transfected with has-miR-146a mimics for 48 h prior to cell lysis detection of the protein expression of TLR2 by western blotting. GAPDH was used as a loading control. (C) Rare C allele upregulated cell culture supernatant production of TNF-α production. The RAW264.7 cells (2×10^5^/well) were plated into 48-well plates and were treated with aggregated Aβ42 (10 *µM*) for 24 h. The supernatants were collected for detection of TNF-α using ELISA kits. Student's t-test was used to analyze the results and P<0.05 was considered to indicate a statistically significant difference (*P<0.05 and.**P<0.01). The results are expressed as the mean ± standard deviation. TLR, Toll-like receptor, TNF, tumor necrosis factor; pri-miR, primary microRNA, Aβ, amyloid β; mmu, mouse mutant.

Since the expression of TLR2 was repressed in the RAW264.7 cells, which are derived from mice (Fig. 4A and B), and the sequences of human miR-146a and mouse miR-146a are identical, the present study detected the biological function of the rare C allele in this cell line. As shown in Fig. 4C, the supernatant levels of TNF-α were reduced by 59.8% (P<0.01) compared with the cells transfected with the empty vector. When transfected with the pri-miR-146a-C vector, the supernatant levels of TNF-α were raised by 71.1% (P<0.05) compared with the pri-miR-146a-C.

## Discussion

The innate immune response and inflammatory signaling are critical for brain homeostasis, neuroprotection and repair (22). If these are overactivated, they produce excess oxygen free radicals, pro-inflammatory cytokines and prostaglandins, subsequently triggering an inflammatory cascade, resulting in neurodegeneration (23).

miR-146a is a negative feedback regulator of the innate immune system and may be important for controlling TLR and cytokine signaling. High expression levels of miR-146a have been identified in various inflammatory diseases, including rheumatoid arthritis (24) and psoriasis (25). Previous investigations have observed that the expression of miR-146a is upregulated in the brain of a mouse model of AD and in the brain tissue of patients with AD (18,19,26). These findings indicated that the overexpression of miR-146a may be used to evaluate the presence and quantify the degree of inflammation in a patient without infection, and may be used as a diagnostic marker for AD. The present study identified the rare C allele of rs2910164, which reduced the expression of miR-146a associated with AD in the Chinese-Han population. This reduced the repressive effect on the expression of its target genes, including TLR2. TLR2 has been confirmed to interact with Aβ42 and is a primary receptor for Aβ42. The roles of TLR2 in Aβ-triggered inflammatory activation and Aβ phagocytosis have been partially elucidated (7). The present study demonstrated that downregulation of miR-146a may be involved in the pathogenesis of AD. Reduced expression of miR-146a may weaken the negative feedback regulation of the inflammatory reaction and increase tissue damage by upregulating the expression of TLR2 during the pathogenesis of AD.

In conclusion, the present study established the first, to the best of our knowledge, association between a polymorphism site and the risk of developing AD in one Chinese-Han population. These findings provide insight into understanding the development of AD and offer a potential approach in the diagnosis and treatment of AD.

## Figures and Tables

**Table I:** Genotype frequencies of rs2910164 in patients and controls and their association with Alzheimer's disease.

Genotype	Patient (n=103) n (%)	Control (n=206) n (%)	Odds ratio (95% CI)	P-value
C	65 (31.550)	97 (0.240)	1.50 (1.030, 2.170)	0.030
G	141 (68.450)	315 (0.76)	0.67 (0.460, 0.970)	
CC	8 (0.078)	4 (0.0190)	4.25 (1.250, 14.47)	
CG	49 (0.480)	89 (0.430)	1.19 (0.740, 1.920)	0.004
GG	46 (0.450)	113 (0.550)	0.66 (0.410, 1.070)	

CI, confidence interval.
